# Dietary Fats and Cognitive Status in Italian Middle-Old Adults

**DOI:** 10.3390/nu15061429

**Published:** 2023-03-16

**Authors:** Walter Currenti, Justyna Godos, Amer M. Alanazi, Giuseppe Lanza, Raffaele Ferri, Filippo Caraci, Giuseppe Grosso, Fabio Galvano, Sabrina Castellano

**Affiliations:** 1 Department of Biomedical and Biotechnological Sciences, University of Catania, 95123 Catania, Italy; 2 Department of Pharmaceutical Chemistry, College of Pharmacy, King Saud University, P.O. Box 2457, Riyadh 11451, Saudi Arabia; 3 Clinical Neurophysiology Research Unit, Oasi Research Institute-IRCCS, 94018 Troina, Italy; 4 Department of Surgery and Medical-Surgical Specialties, University of Catania, 95123 Catania, Italy; 5 Sleep Research Centre, Department of Neurology IC, Oasi Research Institute-IRCCS, 94018 Troina, Italy; 6 Neuropharmacology and Translational Neurosciences Research Unit, Oasi Research Institute-IRCCS, 94018 Troina, Italy; 7 Department of Drug and Health Sciences, University of Catania, 95125 Catania, Italy; 8 Center for Human Nutrition and Mediterranean Foods (NUTREA), University of Catania, 95123 Catania, Italy; 9 Department of Educational Sciences, University of Catania, 95124 Catania, Italy

**Keywords:** dietary fats, short chain fatty acids, cognition, mental health, saturated fats, cognitive impairment, polyunsaturated fatty acids, monounsaturated fatty acids, cognitive decline, nutritional psychiatry

## Abstract

The increase in life expectancy led to a significant rise in the prevalence of age-related neurological diseases, such as cognitive impairment, dementia, and Alzheimer’s disease. Although genetics certainly play a role, nutrition emerged as a key factor in maintaining optimal cognitive function among older adults. Therefore, the study aimed to investigate whether specific categories and subcategories of dietary fats, based on carbon-chain length, are associated with cognitive status in a cohort of 883 Italian participants over the age of 50. Methods: The intake of total, single class of dietary fat, such as saturated fatty acids (SFA), monounsaturated fatty acid (MUFA), and polyunsaturated fatty acid (PUFA), and also single fatty acids grouped according to carbon-chain length, were evaluated by food frequency questionnaires (FFQs). Cognitive health was assessed using the short portable mental status questionnaire (SPMSQ). Results: After adjustment for potential confounding factors subjects with a moderate consumption of both short-chain SFA (for Q2 vs. Q1, OR = 0.23, 95% CI: 0.08, 0.66) and middle-chain SFA specifically lauric acid (C12:0) intake (for Q2 vs. Q1, OR = 0.27, 95% CI: 0.09, 0.77) were less likely to suffer from cognitive impairment. Among single MUFAs, erucic acid (C22:1) intake resulted in an inverse association, in a linear way, with cognitive impairment (for Q4 vs. Q1, OR = 0.04, 95% CI: 0.00, 0.39). Conversely, moderate intake of linoleic acid (C18:2) was associated with cognitive impairment (Q3 vs. Q1, OR = 4.59, 95% CI: 1.51, 13.94). Regarding other PUFAs, individuals consuming moderate intake alpha linolenic acid (C18:3) were less likely to have cognitive impairment (for Q3 vs. Q1, OR = 0.19, 95% CI: 0.06, 0.64). Conclusions: Total SFA intake appeared to be inversely associated with cognitive impairment. Regarding specific subtypes of fatty acids, the results mostly referred to short- and middle-chain SFA. Further studies are needed to validate the results of the present study.

## 1. Introduction

The important rise in life expectancy strongly increased the impact of neurodegenerative diseases related to aging. It is estimated that there are more than 55 million people affected by dementia [1], with 150 million cases predicted by 2050 worldwide [2]. These are alarming figures considering that a non-specifical global decline in cognition was found in approximately 25–50% of the community-dwelling older adults [3]. Progressive loss in memory, difficulties in orientation, reduction in comprehension and judgment are typical features of cognitive decline [4] and usually evolve into pathological diagnosis [5]. To date, despite pharmacological progress, there are no successful therapies to cure cognitive impairment [6]; thus, it is important to understand how to prevent or delay cognitive deterioration. Despite the fact that the onset of cognitive impairment is multifactorial and significantly genetically determined, modifiable risk factors such as lifestyle and nutrition [7,8], including the Western diet characterized by high intake of trans and saturated fatty acids, refined sugars, salt, and ultra-processed foods, were linked to an increased risk of dementia [9]. Conversely, adherence to a plant-based diet such as the Mediterranean diet, rich in unsaturated fats, fibers, and polyphenols, was associated with a lower risk of age-related cognitive decline [10,11]. Regarding single vitamins, observational studies showed an inverse association between higher vitamin C, pyridoxine (B6), folic acid (B9), cobalamin (B12) intakes and cognitive decline [12], probably also affecting the homocysteine metabolism that is, in turn, linked to cognitive impairment and cardiovascular risk [13]. Considering that metabolic syndrome and hyperinsulinemia are associated with worse cognition [14], it is also important to lower insulin and caloric intake; this justifies the interesting suggestion regarding the potential beneficial effects of a ketogenic diet [15] and intermittent fasting on cognitive impairment [16]. Additionally, a vitamin D deficiency may increase the risk of dementia and reduce cognitive performance [17]. Regarding macronutrients intake, dietary fats, and especially saturated fatty acids (SFA), were incriminated for being the main nutritional determinant for cognitive impairment in older adults [18]. On the other hand, both monounsaturated fatty acids (MUFAs) and polyunsaturated fatty acids (PUFAs) may have positive effects toward cognition acting directly on neuronal membrane [19] to the point that their isocaloric substitution to SFAs is usually considered to provide global health benefits [20]. However, recent data showed possible beneficial effects of SFAs on mental health [21,22] due to the different length of the carbon chain that, in turn, determines differential absorption and metabolic effects [23]. Short chain fatty acids are produced by gut microbiota through fermentation of dietary fiber or directly ingested from dairy foods. Interestingly, SCSFAs seem to have several positive effects toward mental health reducing neuroinflammation, regulating the HPA axis and increasing the production of neurotransmitters [22,24]. The study objective was to examine the potential association between cognitive status and specific categories and subcategories of dietary fats based on carbon-chain length in a cohort of middle-aged subjects residing in southern Italy.

## 2. Materials and Methods

### 2.1. Study Population

The MEAL study was a cross-sectional investigation that sought to explore the link between dietary and lifestyle habits of the Mediterranean region and non-communicable diseases (NCDs). The study cohort included 2044 male and female adults who were randomly selected from the major districts of Catania, a city in southern Italy, between 2014 and 2015 (Supplementary Figure S1). Deepening and details about the study protocol were reported elsewhere [25]. Considering that the investigated outcome had a major relevance in the elderly, the analysis was restricted only on participants of 50 years old or older (*n* = 916). Written consent was obtained only after informing everyone about the purposes of the study. The study protocol was approved and reviewed by the relevant ethical committee, and all procedures were conducted in accordance with the World Medical Association’s Declaration of Helsinki (1989).

### 2.2. Data Collection

An expert operator conducted individual-assisted interviews to collect data, which were electronically recorded using tablets. The socio-demographic data collected included age at recruitment, gender, and highest educational degree attained, with educational level classified as (i) low (primary/secondary), (ii) medium (high school), or (iii) high (university). Physical activity was assessed using the international physical activity questionnaire (IPAQ), which comprised five domains that enabled classification of participants into (i) low, (ii) moderate, or (iii) high physical activity categories [26]. Participants were categorized as (i) non-smokers, (ii) ex-smokers, or (iii) current smokers. Lastly, individuals were grouped according to body mass index (BMI) cut-offs, with under/normal weight defined as a BMI < 25 kg/m^2^, overweight as BMI between 25 and 29.9 kg/m^2^, and obese as a BMI ≥ 30 kg/m^2^ [27].

### 2.3. Dietary Assessment

Two food frequency questionnaires (FFQs; a short and a long version) were previously validated in a sample of 178 Sicilian adults living in Sicily, south Italy [28,29]. FFQs contained a total of 110 food items consumed in the last 6 months. The determination of the food ingested, calories introduced, and, in particular, micro- and macro-nutrient intake was calculated using food composition tables of the Research Center for Foods and Nutrition. The reported frequency of consumption in standard portions sizes from FFQs allowed us to obtain, in milliliter or grams, the mean daily consumption of each food. From these data, the total amount of specific fatty acids, taking the values of nutritional food composition tables as reference, was calculated. Adherence to the Mediterranean diet was evaluated through a literature-based score system that assigned positive points for typical foods belonging to Mediterranean habits as legumes, fruits, olive oil, fish, and vegetables, while negative points were assigned for dairy products, meat, and ultra-refined foods. The system contained nine food groups and the score ranged from 0 points (low adherence) to 18 points (high adherence), in order to allow the classification of individuals in tertiles (low, medium, or high adherence to the Mediterranean diet) [30]. After excluding incomplete or unrealistic FFQs (<1000 or >6000 kcal/d), a total of 883 individuals were finally analyzed.

### 2.4. Cognitive Evaluation

The short portable mental status questionnaire (SPMSQ) [31] was used to evaluate cognition in both general and hospitalized populations. This 10-item tool, administered by clinicians in either an office or hospital setting [32], assessed the level of cognitive decline. The tool was helpful for interpreting results, as it provided predetermined classes based on the number of errors: intact (less than 3 errors), mild (3 to 4 errors), moderate (5 to 7 errors), and severe (8 or more errors). In this study, cognitive impairment was defined as more than 2 errors.

### 2.5. Statistical Analysis

Continuous variables were described by means and standard deviations (SDs), while categorical variables were presented as frequencies and percentages. Participants were divided into quartiles based on their total fat intake, and socio-demographic data were compared between groups. Differences in variables were analyzed using the chi-squared test for categorical variables, ANOVA for normally distributed continuous variables, and the Kruskall–Wallis test for non-normally distributed variables. To examine the association between dietary fat intake and cognitive health, energy-adjusted and multivariate logistic regression models were employed. The multivariate model was adjusted for age, sex, BMI, physical activity, educational status, smoking status, and adherence to the Mediterranean diet as an indicator of diet quality, to determine whether the observed associations were independent of these variables. All *p*-values were reported as two-sided and compared to a significance level of 5%. Statistical calculations were performed using SPSS 17 software (SPSS Inc., Chicago, IL, USA).

## 3. Results

A total of 883 participants were finally analyzed. Table 1 showed socio demographic data of the cohort, distributed by quartiles of total dietary fat intake. First, individuals consuming more total fats were younger. A significant difference in the distribution of smoking status and adherence to the Mediterranean diet were found. In particular, in the fourth quartile, there were more former smokers and individuals with moderate adherence to the Mediterranean diet, compared to the lower quartiles. Similar significant differences were found in the distributions of sex and physical activity levels, but without a linear trend. No significant differences between quartiles of dietary fat consumption were observed when considering the educational level and BMI categories.

Table 2 showed the association between total and classes of dietary fats and cognitive status. A multivariate-adjusted analysis revealed a significant inverse association between SFAs and cognitive status in a linear way (for Q4 vs. Q1, odds ratio (OR) = 0.27, 95% CI: 0.09, 0.87). No associations were found between intake of total fats, total MUFAs and PUFAs, and cognitive status.

Table 3 shows the association between specific sub-classes of fats and cognitive status. Interestingly, participants with a moderate consumption of both short-chain saturated fatty acids (SCSFA) (for Q2 vs. Q1, OR = 0.23, 95% CI: 0.08, 0.66) and medium-chain saturated fatty acids (MCSFA), specifically C12:0 (for Q2 vs. Q1, OR = 0.27, 95% CI: 0.09, 0.77), were less likely to have a cognitive impairment. Among single MUFAs, C22:1 intake resulted in an inverse association with cognitive status (Q4 vs. Q1, OR = 0.04, 95% CI: 0.00, 0.39). Conversely, moderate intake of C18:2 was associated with higher odds of having impaired cognition (for Q3 vs. Q1, OR = 4.59, 95% CI: 1.51, 13.94). Regarding other PUFAs, only participants with moderate intake C18:3 were less likely to have a cognitive impairment (for Q3 vs. Q1, OR = 0.19, 95% CI: 0.06, 0.64). No associations were found for other investigated fatty acids and cognitive status.

Table 4 showed the association between individual food sources of fats and cognitive status. Most of the food groups were not associated with the outcome of interest with exception for higher consumption of yogurt (for Q3 vs. Q1, OR = 0.39, 95% CI: 0.19, 0.79) and low intake of sweets and snacks (for Q2 vs. Q1, OR = 0.30, 95% CI: 0.12, 0.71).

## 4. Discussion

In this study, we assessed the possible association relationship between dietary subtype fat intake and cognitive status in a sample of middle-old participants living in a Mediterranean area. Although SFAs have always been considered detrimental toward cognitive health, in our study, after multivariate analysis, higher total SFA intake was associated with lower cognitive impairment. In fact, previous research on rodents showed that a high SFAs diet may affect the hippocampus and prefrontal cortex morphology [33], mainly through a reduced branching and spine density with a consequence on memory loss [34]. Moreover, it appeared that higher SFAs levels increase the concentrations of the protein amyloid beta [35] with a compromission of the blood–brain barrier [36]. Human research was mainly in line with data found in animal models; cross-sectional and longitudinal studies showed that a diet rich in SFAs worsened different cognitive functions; for example, visuospatial learning [37] and prospective and verbal memory performance [38]. Another possible mechanism that links SFAs to cognitive decline may be the rise in cholesterolemia associated with their consumption; in fact, high levels of blood cholesterol play a detrimental role in amyloid beta production and deposition [39]. This hypothesis lost its strength due to new data, showing that SFAs are not the main determinant of LDL cholesterol and cardiovascular risk [21,40], especially in individuals without the APOE ε4 allele that favors the accumulation of intracellular cholesterol [41]. A recent meta-analysis of prospective cohort studies showed higher SFA intake was associated with increased odds of suffering from a cognitive impairment; however, the authors concluded that the results should be interpreted with caution due to the great heterogeneity in the sample size, with regard to the considered cognitive outcomes and dietary subtype of fat [42]. A cohort study with younger participants (average age of 55 years) failed to find a detrimental relationship between high SFAs intake and cognitive decline [43]. Two other cohort studies conducted on older women [44] and women at high vascular risk [45] also showed the absence of detrimental association between SFAs and cognitive decline, leaving the debate open. A possible reason for these disputable results may be due to different subtypes of SFAs mainly ingested from the diet according to the length of their carbon chain that affect their absorption and metabolism [23]. Interestingly, in our cohort, we found that individuals with a higher intake of SCSFA and MCSFA, especially lauric acid (C:12), were less likely to have cognitive impairment. SCSFA may influence cognition firstly regulating the gut-brain-axis. SCFAs were shown to improve gut barrier function, promoting bacterial promoting bacterial diversity [46] and increasing expression of tight junction proteins, which regulate the permeability toward molecules as LPS that may be harmful if pass into the bloodstream [47,48]. SCSFAs activate many types of neurons in the enteric nervous system modulating neurotransmitter release such as serotonin, gamma-aminobutyric acid (GABA), and acetylcholine, which have all been implicated in the regulation of mood and behavior [49]. A recent study showed that supplementation with a mixture of SCFAs increased microbiota diversification and improved cognition and stress in healthy human participants [50]. SCFAs can also influence the production of brain-derived neurotrophic factor (BDNF), a neurotrophin that is involved in neuronal growth and plasticity [51]. In animal models, butyrate directly affects the expression of BDNF in the prefrontal cortex thus improving spatial learning and working memory [52]. Secondarily SCSFAs may influence cognition reducing neuroinflammation binding to G-protein-coupled receptor 43 (GPR43) inhibiting NF-κB signaling [53] and to transform macrophage into anti-inflammatory M2-type [54]. In addition, they have an impact on neuroinflammation by regulating microglia activation, leading to a decrease in the levels of pro-inflammatory cytokines [55]. SCFAs can also protect against oxidative stress that recognized to be a risk factor for mental diseases [56], modulating the activity of nuclear factor erythroid 2-related factor 2 (Nrf2) and the synthesis of antioxidant enzymes such as catalase and superoxide dismutase (SOD) [57]. Finally, SCSFAs may have also effect on mental health through epigenetic regulation; they can inhibit histone deacetylases (HDACs) that in turn leads to increased acetylation of histones and thus changes in gene expression involved in inflammation, synaptic plasticity, and stress response, which are all implicated in the development of mental health disorders [58,59]. A proof that carbon length matter is that long chain saturated fatty acids (LCSFA) as palmitate was found to stimulate inflammation in macrophages and affect microglial and astrocytic signaling pathways [60,61]. Also, MSCSFA as lauric acid may have beneficial effects on cognition reducing the inflammation induced by LPS in microglia [62] and regulating the production of cytokines and neurotrophic factors [63]. In our cohort, a large part of dietary SCSFA-MCSFAs derive from the consumption of dairy products such as yogurt. Interestingly, individuals consuming yogurt have lower odds to manifest cognitive impairment as previously reported in a community-dwelling older adults cohort of the Canadian Longitudinal Study [64]. 

Regarding MUFAs, only the consumption of erucic acid (C22:1) was inversely associated with cognitive impairment in a linear way in our study. Erucic acid is an omega 9 fatty acid derived from vegetable oils that was previously considered toxic from animal research, however new studies on humans reveal that it may enhance cognitive function especially memory [65] interacting with peroxisome proliferator-activated receptors (PPARs), and inhibit elastase and thrombin, thereby modulating neuroinflammation and reducing the levels of pro-inflammatory cytokines [66]. 

Among PUFAs subcategories, we showed an inverse association between alpha linolenic acid (ALA, C18:3) and cognitive impairment while the contrary was found for linoleic acid (LA, C18:2) intake. These results are in line with a recent meta-analysis that reported a beneficial effect of PUFAs especially n-3 on cognitive impairment, dementia and also Alzheimer’s disease [42]. n-3 PUFAs as ALA, which is precursor of DHA and EPA, and was shown to ameliorate learning, semantic [67], spatial [68] and short-term memory [69] in older adults thus preventing cognitive decline [70]. From a mechanistic point of view n-3 PUFAs may affect cognitive health directly increasing membrane fluidity [71], regulating serotonin levels from presynaptic neurons [72] increasing TGF-β1 production [73], and reducing cerebral glutamate [74] which may be neurotoxic [75]. Moreover n-3 PUFAs may act indirectly improving insulin [76], that is a key hormone linked to oxidative stress and neuroinflammation [77]. On the other hand, in line with our results, previous literature showed a possible detrimental role of n-6 PUFAs as LA toward cognitive health. Preclinical studies reported that high intake of LA may induce inflammation into the nervous system through the production of oxidized metabolites [78]. A 12-week dietary trial consists of reducing LA intake from 7% to 2% adding 1.5 g of DHA and EPA, reduced migraine occurrence and ameliorated quality of life in chronic headache patients [79]. Moreover, a lower n-6/n-3 ratio predicts better hippocampus-dependent spatial memory and cognition in older adults [68]. Despite this evidence, the role of LA in cognition remains to be investigated considering that the function of its oxidized metabolites is not yet fully understood and that it has low concentration in the brain [80]. 

Albeit this is the first study in the literature to examine the role of each fat subcategory on cognition, the results may have some limitations. First, it is not possible to determine causal relationship between variables but only association due to the epidemiological study design. Although a multivariate-adjusted logistic regression analysis was done, any additional confounding factors may not have been considered. Another limitation of the study may be related to the use of FFQ for the assessment of dietary intake; in fact, although they are the most widely used tools in epidemiological studies, it is well known that they may potentially over or under-estimate dietary intake due to portion size miscalculation or recall bias. Finally, the evaluation of cognitive health should be carried out in a clinical setting in which a battery of neuropsychological tests can be used rather than just one. Despite it all, SPMSQ is widely used in epidemiological studies and best perform as a screening tool of cognitive decline.

## Figures and Tables

**Table 1:** Background characteristics of the study sample by consumption of total dietary fats (*n* = 883).

	Total Fats				*p*-Value
	Q1 (*n* = 198)	Q2 (*n* = 250)	Q3 (*n* = 242)	Q4 (*n* = 193)	
**Sex, *n* (%)**					0.006
Male	68 (34.3)	103 (41.2)	122 (50.4)	89 (46.1)	
Female	130 (65.7)	147 (58.8)	120 (49.6)	104 (53.9)	
**Age, mean (SD)**	67.1 (10.1)	64.8 (9.1)	64.8 (9.5)	62.8 (9.2)	<0.001
**Educational level, *n* (%)**					0.137
Low	108 (54.5)	116 (46.4)	117 (48.3)	110 (57.0)	
Medium	65 (32.8)	90 (36.0)	76 (31.4)	54 (28.0)	
High	25 (12.6)	44 (17.6)	49 (20.2)	29 (15.0)	
**Smoking status, *n* (%)**					<0.001
Non-smoker	128 (64.6)	157 (62.8)	123 (50.8)	89 (46.1)	
Current smoker	40 (20.2)	52 (20.8)	50 (20.7)	45 (23.3)	
Former smoker	30 (15.2)	41 (16.4)	69 (28.5)	59 (30.6)	
**Physical activity level, *n* (%)**					0.022
Low	66 (33.8)	53 (24.8)	33 (20.1)	44 (25.6)	
Medium	89 (45.6)	99 (45.4)	92 (56.1)	90 (52.3)	
High	40 (20.5)	66 (30.3)	39 (23.8)	38 (22.1)	
**BMI categories, *n* (%)**					0.066
Normal	82 (42.1)	92 (38.0)	74 (33.3)	52 (30.2)	
Overweight	62 (31.8)	102 (42.1)	95 (42.8)	80 (46.5)	
Obese	51 (26.2)	48 (19.2)	53 (23.9)	40 (23.3)	
**Mediterranean diet adherence, *n* (%)**					0.001
Low	119 (60.1)	117 (46.8)	123 (50.8)	82 (42.5)	
Medium	68 (34.3)	97 (38.8)	89 (36.8)	95 (49.2)	
High	11 (5.6)	36 (14.4)	30 (12.4)	16 (8.3)	
Alcohol consumption, *n* (%)					0.002
None	49 (24.7)	57 (22.8)	48 (19.8)	36 (18.7)	
Occasional	120 (60.6)	158 (63.2)	129 (53,3)	108 (56)	
Regular	29 (14.6)	35 (14)	65 (26.9)	49 (25.4)	
**Cognitive impairment, *n* (%)**	35 (17.7)	16 (6.4)	20 (8.3)	11 (5.7)	<0.001

**Table 2:** Association between total and classes of dietary fats and cognitive status in the study sample (*n* = 883).

	OR (95% CI)			
	Q1	Q2	Q3	Q4
**Total fats**				
Energy-adjusted	1	0.53 (0.26, 1.10)	0.96 (0.43, 2.15)	0.81 (0.26, 2.51)
Multivariate-adjusted	1	0.52 (0.25, 1.10)	0.82 (0.36, 1.87)	0.63 (0.19, 2.02)
**Saturated fats**				
Energy-adjusted	1	* 0.45 (0.23, 0.91)	* 0.40 (0.17, 0.95)	0.44 (0.15, 1.30)
Multivariate-adjusted	1	* 0.41 (0.20, 0.83)	* 0.32 (0.13, 0.77)	* 0.27 (0.09, 0.87)
**MUFA**				
Energy-adjusted	1	0.62 (0.30, 1.28)	1.38 (0.59, 3.23)	1.11 (0.35, 3.51)
Multivariate-adjusted	1	0.63 (0.30, 1.30)	1.30 (0.55, 3.07)	0.91 (0.28, 2.95)
**PUFA**				
Energy-adjusted	1	0.50 (0.24, 1.06)	1.00 (0.43, 2.36)	0.72 (0.22, 2.37)
Multivariate-adjusted	1	0.52 (0.25, 1.10)	1.09 (0.46, 2.59)	0.74 (0.22, 2.44)

Multivariate models were adjusted for age, sex, educational level, smoking status, physical activity level, total energy intake, and adherence to the Mediterranean diet. * *p* value < 0.005.

**Table 3:** Association between specific fats and cognitive status in the study sample (*n* = 883).

	OR (95% CI)			
	Q1	Q2	Q3	Q4
**Saturated fats**				
SCSFAs (C4-C10)	1	* 0.23 (0.08, 0.66)	0.35 (0.09, 1.22)	0.60 (0.15, 2.38)
MCSFAs (C12:0)	1	* 0.27 (0.09, 0.77)	0.51 (0.17, 1.47)	0.53 (0.16, 1.76)
C14:0	1	1.86 (0.71, 4.87)	3.61 (0.78, 1.67)	3.52 (0.56, 2.22)
C16:0	1	0.62 (0.18, 2.16)	0.94 (0.13, 7.00)	0.49 (0.03, 6.69)
C18:0	1	0.65 (0.18, 2.30)	0.60 (0.09, 3.63)	0.28 (0.03, 2.88)
C20:0	1	0.59 (0.27, 1.28)	2.28 (0.89, 5.86)	3.67 (0.86, 1.56)
C22:0	1	1.29 (0.63, 2.63)	0.91 (0.36, 2.29)	0.41 (0.10, 1.60)
**MUFA**				
C14:1	1	1.36 (0.68, 2.72)	0.91 (0.38, 2.18)	2.45 (0.89, 6.74)
C16:1	1	0.67 (0.27, 1.62)	0.90 (0.31, 2.65)	0.81 (0.18, 3.65)
C18:1	1	0.76 (0.32, 1.80)	1.86 (0.58, 5.54)	0.64 (0.15, 2.78)
C20:1	1	1.20 (0.56, 2.57)	1.26 (0.39, 4.05)	8.82 (0.91, 8.53)
C22:1	1	* 0.47 (0.22, 0.97)	* 0.04 (0.01, 0.18)	* 0.04 (0.00, 0.39)
**PUFA**				
C18:2	1	2.04 (0.89, 4.65)	4.59 (1.51, 13.94)	2.03 (0.51, 8.05)
C18:3	1	* 0.38 (0.17, 0.85)	* 0.19 (0.06, 0.64)	0.51 (0.16, 1.64)
C20:4	1	0.74 (0.37, 1.51)	0.63 (0.25, 1.60)	0.56 (0.17, 1.80)
C20:5	1	0.68 (0.24, 1.95)	0.34 (0.05, 2.19)	0.88 (0.02, 3.91)
C22:6	1	2.11 (0.05, 84.16)	1.13 (0.03, 40.64)	0.83 (0.03, 22.02)

All analyses were adjusted for age, sex, educational level, smoking status, physical activity level, total energy intake, and adherence to the Mediterranean diet. * *p* value < 0.005.

**Table 4:** Association between food groups and cognitive status in the study sample (*n* = 883).

	OR (95% CI)			
	Q1	Q2	Q3	Q4
Milk	1	1.41 (0.75, 2.65)	0.83 (0.43, 1.62)	-
Yogurt	1	0.78 (0.41, 1.46)	* 0.39 (0.19, 0.79)	-
Cheese	1	1.40 (0.71, 2.78)	0.72 (0.34, 1.55)	1.15 (0.51, 2.61)
Butter	1	0.97 (0.55, 1.68)	0.54 (0.24, 1.22)	-
Fruit and vegetables	1	1.14 (0.57, 2.26)	1.47 (0.73, 2.98)	0.85 (0.36, 1.97)
Sweets and snacks	1	* 0.30 (0.12, 0.71)	1.06 (0.54, 2.07)	0.92 (0.39, 2.18)
Nuts	1	0.91 (0.46, 1.78)	0.66 (0.30, 1.47)	0.91 (0.41, 2.03)
Eggs	1	0.54 (0.22, 1.36)	0.93 (0.43, 2.00)	0.73 (0.34, 1.56)
Meat	1	0.87 (0.43, 1.76)	0.95 (0.46, 1.99)	0.76 (0.37, 1.56)
Processed meat	1	0.80 (0.40, 1.59)	0.98 (0.48, 1.99)	0.73 (0.35, 1.49)
Fish and seafoods	1	0.87 (0.41, 1.85)	0.98 (0.49, 1.95)	1.13 (0.55, 2.34)
Olive oil	1	1.84 (0.78, 4.37)	1.39 (0.61, 3.20)	-
Other vegetable oils	1	1.17 (0.69, 1.97)	-	-

All analyses were adjusted for age, sex, educational level, smoking status, physical activity level, total energy intake, and adherence to the Mediterranean diet. * *p* value < 0.005.

## Data Availability

The data that support the findings of this study are available upon reasonable request.

## Supplementary Materials

The following supporting information can be downloaded at: https://www.mdpi.com/article/10.3390/nu15061429/s1, Figure S1: Study design and recruitment.
