# Supplementary figures and images for: Dietary Fats and Cognitive Status in Italian Middle-Old Adults
Source: Nutrients. 2023 Mar 16;15(6):1429. doi: 10.3390/nu15061429 (PMC10054310; doi:10.3390/nu15061429)

**Figure S1.** Study design and recruitment.

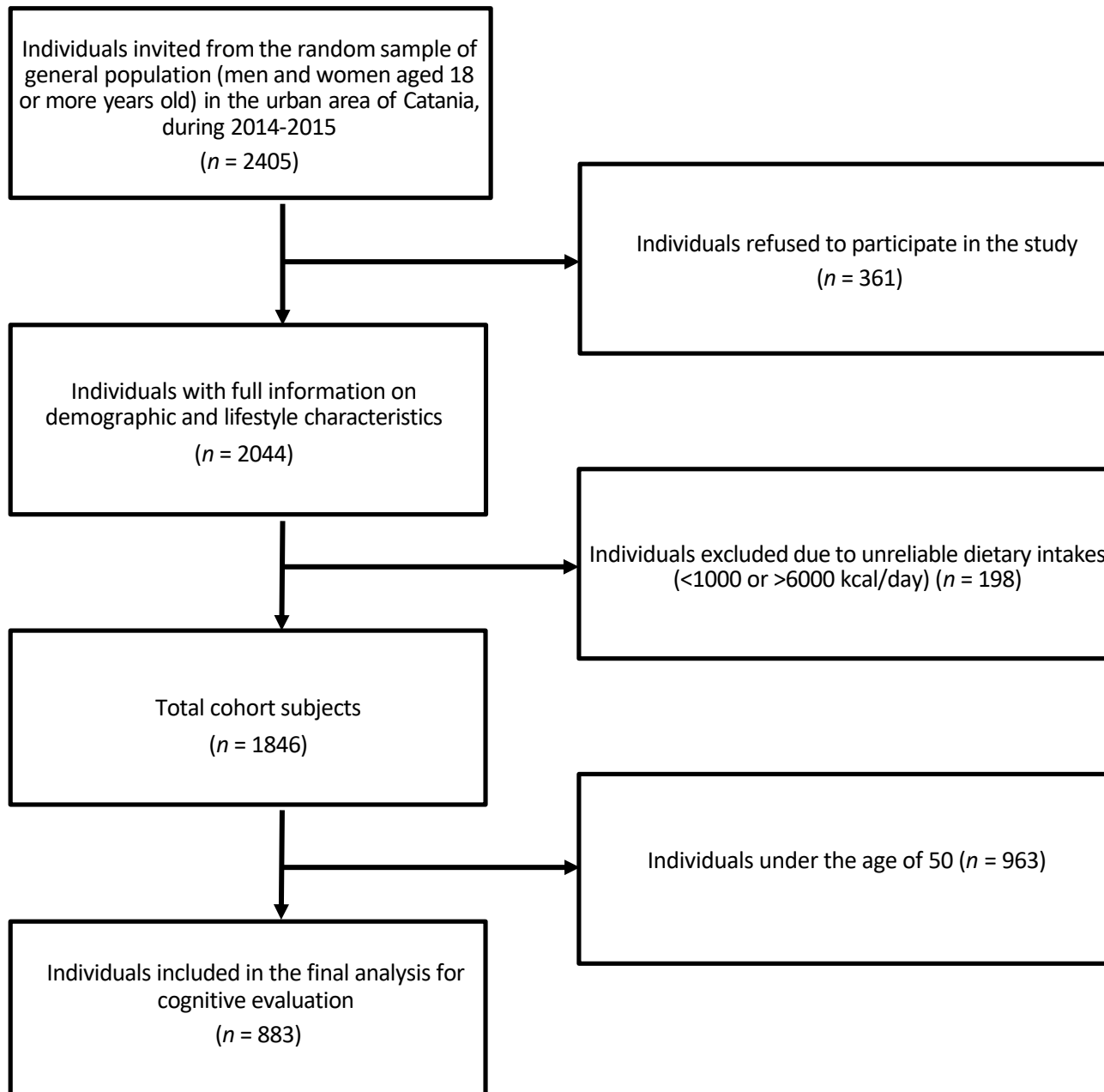

Supplement: Supplementary file 1 [file nutrients-15-01429-s001.zip › nutrients-2209165-supplementary.pdf]
